# Heart Rate Dependence of the Pulmonary Resistance x Compliance (RC) Time and Impact on Right Ventricular Load

**DOI:** 10.1371/journal.pone.0166463

**Published:** 2016-11-18

**Authors:** Thomas S. Metkus, Christopher J. Mullin, E. Wilson Grandin, J. Eduardo Rame, Emmanouil Tampakakis, Steven Hsu, Todd M. Kolb, Rachel Damico, Paul M. Hassoun, David A. Kass, Stephen C. Mathai, Ryan J. Tedford

**Affiliations:** 1 Division of Cardiology, Johns Hopkins University School of Medicine, Baltimore, MD, United States of America; 2 Division of Pulmonary and Critical Care Medicine, Johns Hopkins University School of Medicine, Baltimore, MD, United States of America; 3 Division of Cardiovascular Medicine, University of Pennsylvania, Philadelphia, PA, United States of America; Indiana University, UNITED STATES

## Abstract

**Background:**

The effect of heart rate (HR) and body surface area (BSA) on pulmonary RC time and right ventricular (RV) load is unknown.

**Methods:**

To determine the association of HR and BSA with the pulmonary RC time and measures of RV load, we studied three large patient cohorts including subjects with 1) known or suspected pulmonary arterial hypertension (PAH) (n = 1008), 2) pulmonary hypertension due to left heart disease (n = 468), and 3) end-stage heart failure with reduced ejection fraction (n = 150). To corroborate these associations on an individual patient level, we performed an additional analysis using high-fidelity catheters in 22 patients with PAH undergoing right atrial pacing.

**Results:**

A faster HR inversely correlated with RC time (p<0.01 for all), suggesting augmented RV pulsatile loading. Lower BSA directly correlated with RC time (p<0.05) although the magnitude of this effect was smaller than for HR. With incremental atrial pacing, cardiac output increased and total pulmonary resistance (TPR) fell. However, effective arterial elastance, its mean resistive component (TPR/heart period; 0.60±0.27 vs. 0.79±0.45;p = 0.048), and its pulsatile component (0.27±0.18 vs 0.39±0.28;p = 0.03) all increased at faster HR.

**Conclusion:**

Heart rate and BSA are associated with pulmonary RC time. As heart rate increases, the pulsatile and total load on the RV also increase. This relationship supports a hemodynamic mechanism for adverse effects of tachycardia on the RV.

## Introduction

The product of pulmonary vascular resistance (PVR) and pulmonary arterial compliance (PAC), or RC Time, is the diastolic decay constant of pulmonary artery pressure. For any given resistance, a shorter time constant translates to faster diastolic pressure decay and a higher pulse pressure. Unlike the systemic circulation, prior studies have found that PVR and PAC, the two major components of right ventricular (RV) afterload, depend upon each other and follow an inverse relationship. This coupled relationship results in a constant RC time. Two reasons have been proposed for their relative tight dependence in the pulmonary circulation: 1) most of the vascular compliance is distributed at the lung periphery and therefore the vessels responsible for resistance also account for a significant proportion of the vascular compliance; and 2) elevations in PVR increase pressure within the vascular lumen such that mean pulmonary artery pressure (mPAP) becomes the most important determinant of PAC [1].

Despite this general dependence, individual RC time constants still display variance as recently highlighted by Chemla [2]. We and others have shown that elevations in left heart filling pressures, estimated by pulmonary artery wedge pressure (PAWP), reduce the RC time to account for some of this scatter [3–5]. Other factors that lower the RC time (and hence increase RV pulsatile loading) include increasing age [3], chronic thromboembolic disease [6] and perhaps exercise [7, 8]. Higher mean pulmonary artery pressure (mPAP) is associated with higher RC time [9–11]. With the exception of changes in PAWP, all of these variances are still quite small compared to those measured for the systemic circulation.

In the systemic circulation, higher heart rates (HR) and smaller body size (described using body surface area [BSA]) have been associated with shorter RC times [12, 13]. However, the potential influence of these clinical factors on the pulmonary RC time and right ventricular afterload has not been reported. Given that faster HR is associated with a poor outcome in a broad array of cardiac [14] conditions including pulmonary arterial hypertension [15], we hypothesized that it is also associated with reduced pulmonary RC time reflecting greater RV afterload.

## Methods

We studied four independent and clinically distinct patient cohorts. For cohort A-C, approval for a retrospective analysis of clinically collected data was obtained from the Johns Hopkins and University of Pennsylvania Institutional Review Boards (IRB). For cohort D, after IRB approval for a prospective study, subjects signed written consent to participate.

### Patient Cohorts

To determine the association of HR and BSA with the RC time as well as resistive and pulsatile components of RV afterload, we analyzed three patient cohorts comprised of patients from two institutions.

Cohort A included all subjects (N = 1008) at Johns Hopkins undergoing right heart catheterization between 2000 and 2010 to evaluate for pulmonary hypertension. Patients with a PAWP>15 mmHg were excluded [3].

Cohort B included patients with unexplained cardiomyopathy undergoing endomyocardial biopsy and hemodynamic assessment at Johns Hopkins between 1982 and 1997. Subjects with mPAP≥ 25 mmHg and pulmonary artery wedge pressure (PAWP)>15 mmHg consistent with pulmonary hypertension due to left heart disease (N = 468) were included [16].

Cohort C included patients (N = 150) with end-stage systolic heart failure undergoing RHC prior to implantation of a continuous-flow left ventricular assist device at the Hospital of the University of Pennsylvania between 2008 and 2014.

Next, to assess the influence of increasing heart rate on resistive and pulsatile components as well as total RV afterload at the individual patient level, we performed incremental atrial pacing experiments in a fourth Cohort (D). This cohort was was comprised of 22 prospectively enrolled subjects with pulmonary arterial hypertension (mPAP ≥ 25mmHg and PAWP ≤ 15mmHg) referred for a right heart catheterization (RHC). After consent and standard RHC, the initial right internal jugular sheath was exchanged for a dual-lumen 9F sheath (#406333, St. Jude’s Medical, St. Paul, MN). This enabled simultaneous placement of a 5F pressure-volume catheter (SPC-570-2, Millar, Houston, TX) into the RV and either a 2.4F bipolar pacing wire or 4F quadripolar pacing catheter (Model #D98500H, Edwards, Irvine, CA or Model #401994, St. Jude’s Medical, St. Paul, MN) into the right atrium. The RV volume signal was calibrated based on same day cardiac MRI volume measurements. Pes points were determined from a set of pressure volume loops with varying preload volumes during phase 2 of the Valsalva maneuver, and fit by perpendicular regression to derive the slope of the end-systolic pressure-volume relationship (ESPVR) as previously described [9]. Pes was closely approximated by RVSP, rather than mPAP (S1 Fig).

Atrial pacing, starting at ~80–90 min^-1^ (slightly above the baseline HR), was then increased in 20 min^-1^ increments until either a peak of 140–150 min^-1^ or the HR at which 2^nd^ degree atrioventricular block was observed, whichever came first. Hemodynamic values at rest and during peak pacing HR were compared. Cardiac output (CO) before pacing and during peak pacing were calculated as HRxSV (from the PV loop). Mean pulmonary artery pressure was estimated as right ventricular systolic pressure (RVSP)x0.61+2 as described by Chemla et al [17]. Total pulmonary resistance was calculated as mPAP/CO.

### Hemodynamic measures

All subjects underwent a clinically indicated right heart catheterization (RHC) and had hemodynamic assessment by a heart failure or pulmonary hypertension specialist using a balloon-tipped pulmonary artery catheter. Pressure measurements were recorded at end-expiration. Cardiac output was estimated by the thermodilution method for cohorts A, B and D, and indirect Fick method for cohort C. PAC was calculated as stroke volume divided by pulmonary pulse pressure. Effective arterial elastance (Ea), a lumped measure of afterload that incorporates both resistive and pulsatile components, was determined by dividing end-systolic pressure (Pes) by stroke volume (SV). In the setting of pulmonary hypertension and increased impedance, RV pressure rises throughout ejection peaks near end-systole. Therefore, Pes in cohort A-C was estimated by right ventricular systolic pressure (RVSP) [18]. Pes was directly measured in cohort D.

As described previously in studies of the left ventricle [19, 20], Ea can be algebraically decomposed as the sum of two terms:

- Ea = (mPAP/SV) + (Pes-mPAP)/SV; where the former represents resistive load (mPAP/SV) and the latter represents pulsatile load (Pes-mPAP)/SV
- Furthermore, mPAP/SV can be re-written as TPR (total pulmonary resistance) x HR or TPR/T (with T representing the heart period in seconds). TPR/T shares the same units as Ea (mmHg/mL)
- Therefore, (Pes-mPAP)/SV = Ea − TPR/T

### Statistics

Data are presented as mean (standard deviation). Comparison of continuous variables between quintiles of HR and BSA was performed via T-test or Mann Whitney Rank Sum test as appropriate. The Shapiro-Wilk test was used to test for normality. Comparison of proportions was performed using chi square test. Multiple linear regression analyses and non-linear regressions to fit RC curves were performed using Stata SE 14.0, and are presenting with Beta coefficient (standard error). RC curves were compared by performing log-log transformation and then by ANCOVA. Hemodynamic variables at baseline and at peak-paced heart rate in Cohort D were compared using paired T test or Wilcoxon signed-rank test as appropriate. There was no significant collinearity among the variables assessed by the VIF command. A p-value<0.05 was considered statistically significant.

## Results

**Fig 1:**
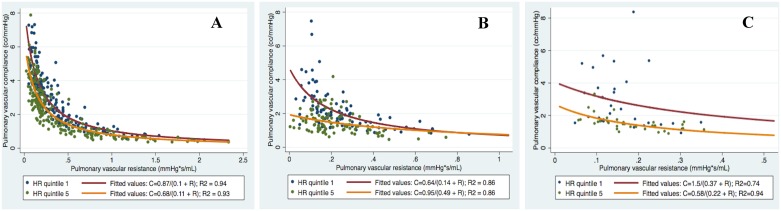
RC curves for quintile 1 and quintile 5 of heart rate in cohorts A-C (p<0.0001 for comparison of curves after log-log transformation).

Baseline characteristics for the 4 independent cohorts are displayed in Table 1 (cohorts A and B have previously been reported [9, 16]). In order to allow for more clear discrimination of variables, we first divided cohorts A-C into quintiles of HR and BSA. Fig 1 displays the RC curves of subjects in cohorts A-C, stratified by highest versus lowest quintile of HR. The RC curve of subjects with the highest quintile of HR was shifted downward compared to the lowest quintile of HR, consistent with lower PAC at any given PVR, a lower RC time, and increased pulsatile RV afterload (p < 0.0001 for all). As expected, effective arterial elastance, a lumped parameter of RV total afterload, was more elevated in the higher heart rate quintiles of cohort A-C. This increase was a consequence of both increased resistive (TPR/T) and pulsatile (Ea-TPR/T) components (Table 2).

**Table 1:** Baseline characteristics for Cohorts A-D.

	Cohort A	Cohort B	Cohort C	Cohort D
	N = 1008	N = 468	N = 150	N = 22
Age (years)	56 (13)	49 (15)	58 (19)	55 (14)
Male sex (%)	383 (38%)	299 (64%)	118 (79%)	5 (23%)
Caucasian race (%)	725 (72%)	285 (61%)	98 (65%)	20 (91%)
Heart rate (bpm)	81 (15)	94 (18)	87 (17)	74 (13)
Body surface area (m2)	1.90 (0.25)	1.95 (0.28)	2.03 (0.31)	1.90 (0.30)
Right atrial pressure (mmHg)	7 (4)	12 (6)	9 (6)	8 (5)
PA systolic pressure (mmHg)	55(27)	54 (12)	51 (12)	60 (22)
PA diastolic pressure (mmHg)	20 (10)	27 (12)	24 (7)	23 (8)
PA mean pressure (mmHg)	33 (16)	36 (8)	33 (8)	37 (13)
Pulmonary artery wedge pressure (mmHg)	10 (3)	26 (6)	21 (7)	9 (4)
Cardiac output (L/min)	5.0 (1.6)	4.0 (1.4)	4.4 (1.2)	4.8 (1.0)
Stroke volume (mL)	63 (22)	44 (17)	53 (19)	65 (14)
Pulmonary vascular resistance (WU)	5.5 (4.9)	2.9 (1.9)	2.9 (1.6)	6.2 (3.8)
Pulmonary vascular resistance (mmHg * s/ mL)	0.33 (0.29)	0.17 (0.11)	0.18 (0.096)	0.37 (0.23)
Pulmonary vascular compliance (mL/mmHg)	2.5 (1.6)	1.8 (1.0)	2.2 (1.3)	2.1 (1.1)
RC time (s)	0.48 (0.17)	0.35 (0.19)	0.33 (0.19)	0.62 (0.15)

Data is displayed as mean (SD) or N (percent). PA = Pulmonary artery.

**Table 2:** Pulsatile, resistive, and total RV load in heart rate quintile 1 versus 5 across Cohorts A-C.

	Heart Rate Quintile 1	Heart Rate Quintile 5	P
**COHORT A**			
Ea (mmHg/mL)	0.73 ± 0.48	1.27 ± 1.02	<0.0001
TPR (mmHg*sec/mL)	0.42 ± 0.26	0.47 ± 0.39	0.54
Heart period, T (sec)	0.98 ± 0.10	0.60 ± 0.05	<0.001
TPR/T (mmHg/mL)	0.43 ± 0.28	0.80 ± 0.64	<0.001
Ea—TPR/T (mmHg/mL)	0.30 ± 0.21	0.48 ± 0.40	<0.001
**COHORT B**			
Ea (mmHg/mL)	1.09 ± .44	1.76 ± 0.66	<0.001
TPR (mmHg*sec/mL)	0.63 ± 0.26	0.61 ± 0.23	0.91
Heart period, T (sec)	0.88 ± 0.10	0.51 ± 0.04	<0.001
TPR/T (mmHg/mL)	0.72 ± 0.30	1.21 ± 0.46	<0.001
Ea—TPR/T (mmHg/mL)	0.38 ± 0.17	0.55 ± 0.25	<0.001
**COHORT C**			
Ea (mmHg/mL)	0.82 ± 0.39	1.46 ± 0.41	<0.0001
TPR (mmHg*sec/mL)	0.46 ± 0.20	0.52 ± 0.17	0.09
Heart period, T (sec)	0.91 ± 0.08	0.54 ± 0.05	<0.001
TPR/T (mmHg/mL)	0.51 ± 0.24	1.00 ± 0.34	<0.001
Ea—TPR/T (mmHg/mL)	0.31 ± 0.03	0.46 ± 0.13	<0.001

Ea = Effective arterial elastance, TPR = total pulmonary resistance, HR = heart rate

**Fig 2:**
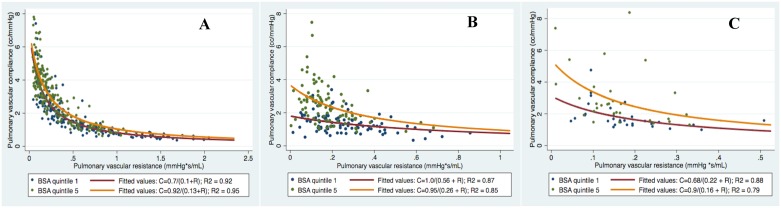
RC curves for quintile 1 and quintile 5 of body-surface area in cohorts A-C (p<0.0001 for comparison of curves after log-log transformation).

Fig 2 displays the RC curves of subjects with highest vs lowest quintile of BSA. The RC curves of subjects with the lowest quintile of BSA was shifted downward compared to the highest quintile of BSA (Fig 2, panels A-C), also consistent with increased pulsatile RV afterload (p < 0.0001 for all). S1–S3 Tables compares the demographic and hemodynamic variables between lowest and highest quintiles of HR and BSA for each of Cohort A, B, and C. PA diastolic pressure was higher in the highest quintile of HR compared to the lowest quintile in all cohorts (p<0.01 for all), without a significant decline in systolic pulmonary artery pressure.

In order to control for the effects of other known determinants of the RC time, we next performed multiple linear regression modeling in Cohorts A-C forcing HR, mPAP, PAWP, BSA, and age, race and sex into the model as covariates. Table 3 summarizes these results. Race and sex did not impact RC time. Increasing HR was significantly associated with lower RC time in cohorts A-C (p<0.001 in all). Lower BSA was also associated with lower RC time in all cohorts (p<0.05 in all). mPAP and PAWP were strong determinants of RC time consistent with prior data [3]. In cohort A where PAWP is constrained between 0-15mmHg, the effect of HR on the RC time was perhaps slightly more robust than the effect of PAWP. However, in Cohorts B and C, both of which are comprised of patients with left heart failure, the PAWP effect on RC time was more substantial. The BSA effect was more modest than that of PAWP or HR and was comparable to the effect of age.

**Table 3:** Multiple linear regression models for determinants of RC Time in Cohorts A-C.

	Cohort A		Cohort B		Cohort C	
	β (SE)	P value	β (SE)	P value	β (SE)	P value
Age	-0.069 (0.0003)	0.006	-0.082 (0.0004)	0.01	-0.019 (0.0009)	0.9
Mean PA Pressure	0.57 (0.0003)	0.0001	0.85 (0.001)	0.0001	0.71 (0.002)	0.0001
PA Wedge Pressure	-0.3 (0.002)	0.0001	-0.96 (0.001)	0.0001	-0.9 (0.003)	0.0001
Heart rate	-0.36 (0.0003)	0.0001	-0.25 (0.0003)	0.0001	-0.19 (0.0008)	0.006
Body-surface area	0.059 (0.02)	0.04	0.89 (0.02)	0.01	0.23 (0.03)	0.0001
Caucasian race	0.0074 (0.009)	0.3	0.19 (0.01)	0.5	-0.0097 (0.02)	0.9
Male sex	0.15 (0.01)	0.5	0.018 (.1)	0.6	0.059 (0.03)	0.3

PA = Pulmonary artery.

In order to test the effects of increasing HR within an individual patient, we performed incremental right atrial pacing. Table 4 demonstrates hemodynamics at baseline and at peak pacing-induced tachycardia in the Cohort D subjects. Of this group, 8 patients had idiopathic PAH, 12 had PAH associated with systemic sclerosis, and 2 had PAH associated with systemic sclerosis and interstitial lung disease. Heart rate increased from 75 ± 13 beats per minute (bpm) at baseline to 134±10 bpm during peak atrial pacing (p = 0.0001). This was associated with a decline in RV systolic pressure (58 ± 22 mmHg to 50 ± 17 mmHg; p = 0.002). Although stroke volume also decreased (67 ± 17 mL to 48 ± 15 mL; p = 0.0001), cardiac output increased from 4.9 ± 1.0 to 6.4 ± 2.2 L/min (p = 0.002) and total pulmonary resistance (TPR) fell during pacing (8.1 ± 3.8 WU at baseline vs. 6.0 ± 3.6 WU at peak heart rate, p = 0.013). Ea increased from 0.87 ± 0.42 to 1.2 ± 0.73 mmHg/mL (p = 0.027) as did the resistive (TPR/T) load: 0.60±0.27 vs 0.79±0.45; p = 0.048. The pulsatile load component of Ea was also higher during pacing tachycardia (0.27 ± 0.18 vs 0.39 ± 0.28, p = 0.03) similar to observations from cohort A-C.

**Table 4:** Hemodynamics at baseline and peak paced heart rate in Cohort D.

	Baseline	Peak pacing rate	P
	Mean (SD)	Mean (SD)	
Heart rate (bpm)	75 (13)	134 (10)	0.0001
Heart period (T, in seconds)	0.83 (0.14)	0.45 (0.03)	0.001
RV systolic pressure (mmHg)	58 (22)	50 (17)	0.002
Mean PA pressure (mmHg)	38 (14)	33 (10)	0.002
Cardiac output (L/min)	4.9 (1.0)	6.4 (2.2)	0.002
Stroke volume (mL)	67 (17)	48 (15)	0.0001
Ea (mmHg/mL)	0.9 (0.4)	1.2 (0.7)	0.03
Total pulmonary resistance (WU)	8.1 (3.8)	6.0 (3.6)	0.01
Resistive load (TPR/T, mmHg/mL)	0.60 (0.27)	0.79 (0.45)	0.048
Pulsatile load (Ea-TPR/T, mmHg/mL)	0.27 (0.18)	0.39 (0.28)	0.03

Data shown as mean (SD).

## Discussion

We demonstrate across clinically distinct patient cohorts that HR and BSA are both associated with the pulmonary RC time and may account for some of the “scatter” previously reported. Thus, the notion of a “fixed” RC time in the pulmonary circulation should be modified by at least two additional factors. The magnitude of effect of HR on the RC time is substantial and comparable to the effects of mPAP and PAWP. We also show heart rate is increased, total RV load increases, including at the individual patient level. The increase in RV load at faster heart rates is mediated not only via the HR effect on resistive load (TPR/T) but also through absolute increases in pulsatile RV afterload.

When RC time is calculated as PVR x PAC, it can be simplified to (mPAP—PAWP)/(HR x pulmonary pulse pressure) because stroke volume cancels out. Therefore, as HR increases and the diastolic time shortens, the pulmonary pulse pressure must decrease and/or mPAP rise in a proportional manner if the RC time is to remain constant. However, in our cohorts, the changes in pulse pressure and mPAP do not fully offset the increase in HR, and the RC time declined. A lower RC time would suggest that at similar levels of resistive load, tachycardia leads to increased pulsatile loading of the right ventricle, and this may have important clinical implications for patients with pulmonary hypertension. Resting HR is an independent predictor of adverse outcome in PAH, likely in part as a reflection of underlying RV function [15]. Although increases in heart rate may be compensating for a lower stroke volume or driven by excess adrenergic activity, our findings suggest this compensatory mechanism could lead to higher RV load. Although some pre-clinical studies and pilot data using beta-blockers in PAH have appeared promising [21–23], recent retrospective propensity-matched analyses have shown no impact on survival [24, 25]. Future clinical trials with beta-blockers or other heart rate slowing agents in PAH may further shed light on this issue. It is important to note that although afterload increased with pacing in our PAH cohort (cohort D) and RV stroke volume declined, overall cardiac output (HRxSV) actually increased during pacing. This may speak to the dependence on heart rate to augment cardiac output in individuals with PAH, and this potential HR dependence needs to be considered carefully in any future studies of heart rate slowing agents in this population. Recent studies have indicated that RV load increases during exercise, even in normal controls [7]. Our findings would suggest that tachycardia induced by exercise accounts for at least some of this increased load.

The mechanism of increasing heart rate leading to a lower RC time is less clear. One possibility is that as the diastolic period decreases (with increasing heart rate), the pulmonary circuit is unable to completely discharge the prior stroke volume (incomplete Windkessel function), and compliance decreases. Indeed, in cohorts A-C, we found an increase in diastolic pulmonary pressure without significant changes in PAWP or pulmonary pulse pressure, the latter of which should fall as stroke volume decreases. MRI studies demonstrating that MRI-measured pulmonary artery distensibility- an index incorporating the difference between systolic and diastolic pulmonary artery diameter- is correlated with response to inhaled vasodilators, outcome, and functional class in pulmonary hypertension [26, 27]. MRI measured PA distensibility also correlates with pulmonary arterial compliance [28]. These measures may not only help confirm what we have witnessed invasively but additionally may be about to tease out if the pulmonary circulation is unable to fully discharge the prior stroke volume with increasing heart rate. In a study of 16 mitral stenosis subjects, Laskey and Kussmaul found that increasing HR (via pacing) led to increasing pulse wave velocity and earlier arrival of reflective waves seemingly in keeping with our findings [29]. However, when controlling for ejection time, the reflective waves actually arrived later in the systolic period (in relative terms) and this was associated with a decrease in pulsatile hydraulic power as measured by impedance [29]. Measurements of pulse wave velocity and changes in impedance with increasing heart rate should be considered as part of ongoing research efforts. Alternatively, simplified equations for resistance, compliance, and the RC time may not reflect true physiologic values [2]. Ea (Pes/SV) is known to be heart rate dependent and estimating compliance as SV/PP, which is mathematically similar, could be influenced by heart rate even if ‘true’ compliance is not. Nonetheless, we show that elements of pulsatile loading increase, even when accounting for the increases in resistive load.

We also found that a lower body surface area was associated with a lower pulmonary RC time. The systemic RC time has been shown to increase with increasing body size across mammals [12]. Lung size, and hence the number and size of compliance vessels in the lung (blood storage capacity), may also be expected to increase with increasing BSA, although at least one prior study found no correlation between BSA and pulmonary blood volume [30]. The proximal pulmonary artery contribution accounts for approximately 20% of PAC [31], and therefore it is conceivable that larger conduit vessels associated with larger body size could potentially contribute modestly to PAC. This finding is consistent with prior studies demonstrating that proximal pulmonary artery obstruction contributes modestly to reduced RC time [6, 32]. Overall, the effect of BSA on the RC time appeared modest, and comparable in magnitude to the effects of aging, suggesting the effect may indeed be a consequence of the proximal vessel size. If the effect of BSA is confirmed in future studies, it could imply that markers of pulmonary vascular load should be scaled to body-size, especially in children where the effect is likely more dramatic.

Limitations of our study include the fact that, in cohorts A through C, heart rate was not an independently controlled variable, but an observational variable made in each patient. This could introduce confounding factors, for example, subjects with higher HR tend to have stiffer pulmonary vasculatures. The cohorts consist of patients with varied clinical characteristics, making comparison of magnitude of effect challenging. It is also possible that faster HR resulted in some overestimation of PA pulse pressures due to fluid filled catheter underdamping. This is unlikely given the frequency range we are describing, but cannot be ruled out. Further, in Cohort D, HR was a true independent variable in each patients, and high fidelity catheters were used, yet a similar result was obtained. In this cohort, only RV pressures were available, so that mPAP had to be estimated and pulse pressure (and hence PAC) could not be determined. Therefore, calculated mPAP may underestimate the pulsatile loading in cohort D and while we witnessed absolute increases in resistive and pulsatile load, it is not possible to truly assess the relative contributions of each. Finally, PAC is clearly a lumped simplification for compliance, and while it has been validated against 3-element Windkessel model calculations, some of this theory has been debated [2].

In conclusion, we demonstrate that HR and BSA are both associated with the pulmonary RC time. As heart rate is increased, pulsatile and total load on the right ventricle also increases. This relationship may suggest a pathophysiologic mechanism for adverse effects of tachycardia on right ventricular afterload.

## Supporting Information

S1 Fig Linear relationship between end-systolic pressure (Pes) derived from multi-beat pressure-volume analysis and right ventricular systolic pressure (RVSP) (panel A) and mean pulmonary artery pressure (mPAP) (panel B). Estimating Pes as mPAP leads to significant underestimation in these subjects with pulmonary arterial hypertension.
(TIF)

S1 Table Clinical and demographic profile of Cohort A stratified by first versus fifth quintile of HR and BSA. Data shown as mean (SD) and N (%). PA = Pulmonary Artery.
(DOCX)

S2 Table Clinical and demographic profile of Cohort B stratified by first versus fifth quintile of HR and BSA. Data shown as mean (SD) and N (%). PA = Pulmonary Artery.
(DOCX)

S3 Table Clinical and demographic profile of Cohort C stratified by first versus fifth quintile of HR and BSA. Data shown as mean (SD) and N (%). PA = Pulmonary Artery.
(DOCX)
