# Supplementary figures and images for: Heart Rate Dependence of the Pulmonary Resistance x Compliance (RC) Time and Impact on Right Ventricular Load
Source: PLoS One. 2016 Nov 18;11(11):e0166463. doi: 10.1371/journal.pone.0166463 (PMC5115737; doi:10.1371/journal.pone.0166463)

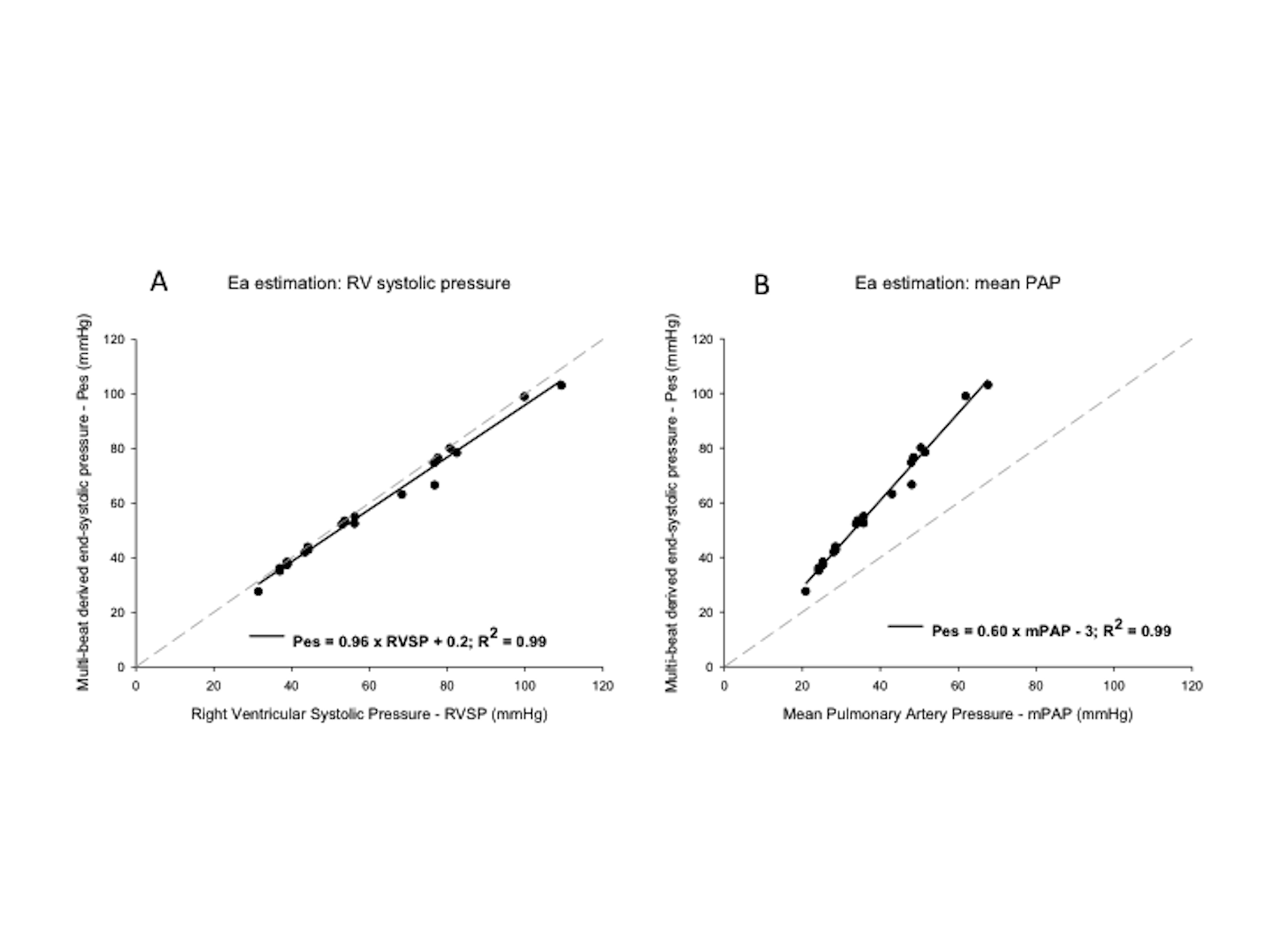

Supplement: S1 Fig — Estimating Pes as mPAP leads to significant underestimation in these subjects with pulmonary arterial hypertension. (TIF) [file pone.0166463.s001.tif]
