# Supplementary material for: Heart Rate Dependence of the Pulmonary Resistance x Compliance (RC) Time and Impact on Right Ventricular Load
Source: PLoS One. 2016 Nov 18;11(11):e0166463. doi: 10.1371/journal.pone.0166463 (PMC5115737; doi:10.1371/journal.pone.0166463)
Supplement: S1 Table — Data shown as mean (SD) and N (%). PA = Pulmonary Artery. (DOCX) [file pone.0166463.s002.docx]

**S1 Table:** Clinical and demographic profile of Cohort A stratified by first versus fifth quintile of HR and BSA.

Data shown as mean (SD) and N (%). PA = Pulmonary Artery.
